# Communicating ocean and human health connections: An agenda for research and practice

**DOI:** 10.3389/fpubh.2022.1033905

**Published:** 2022-12-02

**Authors:** Marcus B. Reamer

**Affiliations:** Department of Environmental Science and Policy, Rosenstiel School of Marine, Atmospheric, and Earth Science, University of Miami, Coral Gables, FL, United States

**Keywords:** environmental communication, ocean and human health, marine ecosystem, coastal communities, interdisciplinary, planetary health

## Abstract

The emergence of ocean and human health (OHH) science as a distinct scholarly discipline has led to increased research outputs from experts in both the natural and social sciences. Formal research on communication strategies, messaging, and campaigns related to OHH science remains limited despite its importance as part of the social processes that can make knowledge actionable. When utilized to communicate visible, local issues for targeting audiences, OHH themes hold the potential to motivate action in pursuit of solutions to environmental challenges, supplementing efforts to address large-scale, abstract, or politicized issues such as ocean acidification or climate change. Probing peer-reviewed literature from relevant areas of study, this review article outlines and reveals associations between society and the quality of coastal and marine ecosystems, as well as key themes, concepts, and findings in OHH science and environmental communication. Recommendations for future work concerning effective ocean and human health science communication are provided, creating a platform for innovative scholarship, evidence-based practice, and novel collaboration across disciplines.

## Introduction

In 1999, the U.S. National Research Council (NRC) published a report, “From Monsoons to Microbes: Understanding the Ocean's Role in Human Health,” outlining data gaps and priority areas for scientific research on the relationships between the quality of aquatic systems and human health. In it, the NRC's Committee on the Ocean's Role in Human Health prioritized several areas of research: the contamination of marine waters and seafood species by microbes and chemicals; natural products that were or could be derived from marine ecosystems; the effects of harmful algal blooms; and global environmental change (1). It was with this report that ocean and human health science (OHH) was formalized as a distinct priority for environmental scholarship and policy (2–5). By 2004, the National Institute of Environmental Health Studies under the U.S. National Institutes of Health partnered with the National Science Foundation to establish the Centers for Ocean and Human Health, through which these institutions support interdisciplinary research, collaboration, and innovation (6). Today, a global community of researchers, environmental organizations, and communities representing a wide range of expertise and experiences have contributed to a body of literature that demonstrates linkages between ecological systems and the determinants of human health and wellbeing (7, 8), an approach that can effectively build public support for policy action to address environmental problems (9).

As research and media coverage of climate change and human health connections with the environment have increased over the last decade or so, marine conservation topics–including ocean and human health–have been largely overlooked (10–12). Still in the early years of the United Nations' Decade of Ocean Science for Sustainable Development (13) and in the absence of an international Ocean Treaty, a unique window of opportunity exists to invite new stakeholders and perspectives into ocean restoration and sustainability by strategically connecting aquatic ecosystems to human health. Such an approach may be particularly useful for engaging audiences that are doubtful or dismissive of climate change and building broad support for pro-environmental actions at the local level. With a focus on strategic environmental communication, this review directly answers calls from thought leaders in ocean science who encourage interdisciplinary and cross-sector collaborations as a way to make findings from OHH research actionable (7, 8, 14–18) and to improve ocean literacy and marine citizenship (19). This review also explores and expands upon advances in environmental communication, drawing from literature that has demonstrated the potential benefits of using public health outcomes to make environmental problems important and relevant to select audience groups, particularly residents of and governments representing coastal communities, as well as health professionals and researchers (20–25). An agenda for research and practice is provided to support existing efforts and encourage future work at the intersection of OHH science and environmental communication.

## The science of ocean and human health

Originally a niche area of scholarly interest for marine scientists, research in OHH science over the past several decades has created a global meta-discipline that is inclusive of knowledge and perspectives across the natural sciences, social sciences, and the humanities (5, 16). Once narrowly focused on measuring and describing hazards and risks, OHH science has expanded considerably, taking a multidimensional approach to understanding complex socioecological systems, exploring the benefits to health and overall wellness provided by ocean resources, and connecting local changes to global challenges (2). Its broad scope and acknowledgment of the complexities and interconnectedness of aquatic systems is both a strength and a limitation for the field.

The literature in OHH science is expansive, covering a wide range of topics and geographic areas (Table 1). Some experts have categorized this body of literature using themes [e.g., (2, 151–153)], though a standard set of categories has not yet been agreed upon. Due to the multi- and interdisciplinary nature of OHH scholarship, there are innumerable ways to sort research in the field and identify priorities for action. To date, there is no peer-reviewed journal dedicated to publishing OHH literature, though special editions of marine science and environmental journals dedicated to the topic have been published. Several persistent challenges in OHH science have limited the development and implementation of “adequate societal response[s]” to identified challenges and opportunities [(16), p. 557]. One is the difficulty involved in defining, operationalizing, and messaging ocean health (16, 154). Another is that the hazards, benefits, and decisions associated with ocean resources are not equally distributed or experienced by people due to variations in ecosystems and social inequalities (155–158). And a third challenge is making ocean issues–which, to many people, feel distant, too large, and unfamiliar (159)–accessible so that people feel they are both relevant and important enough to act on (160–165).

**Table 1:** Examples of topics in OHH science and relevant sources.

**Topic or category**	**Description**	**Related literature**
Physical and chemical processes	Research on physical and chemical processes in the ocean and atmosphere that transport nutrients and pollutants, affects air quality, or changes the composition of seawater	(26–32)
Sentinel species	The use of animal behavior, biomarkers, or mortality events to predict or observe changes in environmental quality. Marine mammals, birds, fish, invertebrates, and even cats have been documented as sentinels for aquatic systems	(33–49)
Seafood nutrition, risks, and food security	Research on the possible health benefits and risks related to seafood consumption, observations of changing seafood nutritional composition related to ecological changes, and the social wellbeing associated with food security and economic opportunity provided by fisheries	(50–69)
Transportation and offshore emissions	The effects of offshore emissions and management efforts on local air quality and health	(70–81)
Disease, injury, and pharmaceuticals	The ocean's role in communicable and non-communicable disease, physical injury and death, and the potential use of marine resources in the development of pharmaceuticals	(82–93)
Pollutant exposure	The effects of physical and chemical pollutant exposure on the human body	(94–112)
Natural disasters, catastrophes, and community resilience	The health-related after effects of natural disasters and catastrophes, including resilience and recovery	(113–122)
Psychological, spiritual, and community health	Benefits or threats to individual and community wellbeing as the result of direct or indirect interactions with the ocean or other natural spaces or as the result of policy decisions pertaining to aquatic resources	(123–140)
Early detection and warning systems	The use of data, expert opinion, and technology to detect changes in local environments and deploy early warnings in affected areas to prevent or minimize harms to individual, community, or public health	(120, 141–150)

The concept of human health is not clearly or simply defined due to the multidimensional nature of health determinants and outcomes, which is why the exact definition of health continues to be debated by experts in the medical and public health fields who hold a diverse range of perspectives (166–169). Ocean health, then, is even more challenging to define. Since the global ocean is not an organism, it can be neither healthy nor sick. This makes the concept of ocean health more of a metaphor than anything else, the meaning behind which experts and advocates have yet to agree upon, in large part due to differing values and beliefs (154, 170). For some, a healthy ocean is one that is productive and sustainable and for others it is an ocean that is pristine, restored to near-historical conditions and showing few signs of human influence. With multiple definitions, variations in expert perspectives, and the ways human health and ocean health are viewed by non-expert audiences, emphasizing connections between these two complex concepts can be quite difficult.

If defining ocean health and human health is challenging, so too is operationalizing, measuring, and acting to achieve it (16, 154). One notable effort to quantitatively measure the health of interconnected human-ocean systems is the Ocean Health Index (OHI) (153). This integrated assessment was designed to measure progress toward ocean health initiatives using inputs related to ten categories that capture “most” of the topics that are of concern to citizens, policy makers, and resource managers [(153), p. 616]. Rather than prescribing a score for the entire ocean, the OHI is best applied to countries or regions [e.g., (171, 172); (170, 173–175)] since “all goals are judged against reference points that describe what is possible or desirable in a particular place” [(153), p. 618]. The OHI's outputs are best and most often used for assessment of current conditions, though it can be used longitudinally or to simulate the consequences of decisions or actions (153). It is not designed to model future conditions or make predictions. The outputs of the OHI can also be used to support public outreach and education, to inform policy decisions, and to identify areas for future research (153).

In their proposed model of operationalized ocean health, Franke et al. (16) argue for a more holistic approach to research and ocean governance that facilitates transdisciplinarity, cross-sector collaboration, and integration. This, they argue, creates a strong foundation for the restoration and maintenance of productive and sustainable marine ecosystems that can also provide human health benefits or mitigate risks. An integral part of this proposed framework is human communication in its many forms, which can facilitate novel research, train scholars in the language of multiple disciplines, educate audiences on ecological challenges and their implications for society, collect and co-produce knowledge based on scientific observation and lived experiences, engage stakeholders in decision-making processes, or to generate support for proposed actions (16).

As is the case with other issues in the environment, the causes and consequences of diminished ocean health are socially disproportionate as environmental hazards, benefits, narratives, and decision outcomes are not distributed or experienced equally throughout society (158, 176–179). The nations, communities, and individuals who do the least damage or that do not overexploit resources often experience disproportionately harmful outcomes that are caused or made worse by the actions of larger or more powerful groups (69, 180, 181). As such, issues in ocean and human health are as much issues of equality and justice as they are of public health and ecology; both academics and practitioners have a responsibility to advance solutions that consider the politics of environmental decision-making (155), intended and unintended outcomes, and advocate for communities that may be overlooked or excluded from these processes.

Already marginalized communities are more likely to experience the worst health outcomes, stand to gain the least from environmental or social benefits (182, 183), are more likely to be excluded from or overlooked in planning processes (184–186), are more likely to be underrepresented or misrepresented in research and publication (187–191), and are at risk of losing the most in terms of culture and quality of life as a result of their natural resources being “appropriated, developed, degraded or destroyed” [(192), p. 369]. Some of the more visible examples of environmental inequalities include the effects of commercial overfishing and injustices involved with aquaculture (69, 193, 194), the effects of reduced access to sea ice by Indigenous communities in Arctic and Subarctic regions (195), and increased exposure to pollutants by lower-income or marginalized communities (196). Not as visible or clearly connected, conservation-oriented decision making and policy implementation can also affect human health, as Loring (123) found in reviewing the longer-term social outcomes of a voter-supported commercial fishing gear ban in Florida waters. The harms commercial fishers and their families experienced were felt well beyond the immediate economic effects of the gear ban, which had mixed ecological results for fishery stocks. Individual psychological health suffered, especially in women (197), and a collective trauma in some of Florida's commercial fishing communities has been observed, which appears to be the result of economic insecurity and being politically villainized within their own communities (123).

While OHH scholarship has advanced considerably over the past several decades, it is not enough to simply identify problems in aquatic ecosystems and link them to human health and wellbeing. Making this knowledge actionable and addressing the persistent challenges in the field requires investment and expertise in communication as a way to share knowledge and build support. With this in mind, there are opportunities for involvement from scholars, practitioners, policymakers, and interested individuals.

## Environmental psychology and communication

Communication is an important and determining factor in the success or failure of efforts in marine conservation (11, 198). This is because human perceptions of and relationships to the natural world are the direct product of communicative and cognitive processes that are involved with the social construction of reality. Factors like emotion, cognitive bias, worldview, life experience, culture, social norms, mass media, and politics can each shape how individuals and groups receive and assimilate information and respond to environmental problems (155, 199–202). As a result, there are “social-cognitive challenges” [(162), p. 2] and externalities like misinformation (203) and media selection biases (204) that experts will inevitably encounter as they try to use facts to inform policy and management decisions (205, 206). To effectively overcome these challenges requires an understanding of individual thinking, social behavior, and institutions, which are well documented in interdisciplinary literature in fields like environmental psychology and environmental communication (207–212).

Like OHH science, environmental communication (EC) has been around for some time but its adoption as a distinct area of academic research and practice is much more recent, appearing in literature in the 1980s and formalizing in the early 2000s (213, 214). EC is a broad term for an interdisciplinary field and its subfields dedicated to solving complex environmental problems by exploring human relationships with the biosphere and encouraging change at the individual, community, organizational, institutional, national, and international levels. EC occurs at the intersection of the life sciences, the social sciences, and the humanities, is inclusive of communication ranging from the interpersonal to the mass mediated, is place-based, embraces cultural and linguistic variabilities, and takes place in the public sphere (202, 215–217). While uniquely dedicated to topics in the environment, EC has synergies with other topically-focused forms of communication such as science, risk, and health communication, which can create opportunities for collaboration, shared learning, and innovation (218). In practical settings, EC plays a critical role in determining the success or failure of sustainability efforts (219) and scholarship can provide practitioners with valuable feedback and evidence to guide their future work (33). EC also has explicitly stated objectives that differentiate it from other forms of science and communication practice.

Fundamentally a crisis discipline (220), EC has an “ethical obligation” to help society see, make sense of, and act on environmental problems, and to equip those affected by changes in the environment with the tools they need to participate in decision-making that affects their health and wellbeing or that of their communities [(221), p. 5]. EC also carries with it a duty to exhibit an ethic of care in pursuit of environmental justice and to acknowledge the interdependence of human and non-human systems in the development and promotion of solutions to environmental problems (222). To achieve these objectives, it is important to acknowledge that one size does not fit all in communication as ecological systems are socially constructed places that hold different meanings for individuals and groups (215, 223). It is also important to design research that incorporates members, organizations, and institutions from affected communities, also known as engaged scholarship (224). The use of EC as an active component of social change efforts sometimes runs counter to a tradition of objectivity in science and by scientists, which can create barriers to participation for trained scientists or other experts who have not received training in communication or policy as part of their professional lives (225–229).

EC literature is inclusive of topics across disciplines, geographies, and sectors, though journals tend to emphasize research on climate change communication and use it as an umbrella subject (162, 213). There are, of course, advantages and disadvantages to doing so. One of the most identifiable limitations is the exclusion of topics not clearly or directly related to climate change, which creates a risk of obscuring those topics from public view entirely (202). Additionally, climate science and policy have become politically polarized topics in some nations, with coordinated messaging from industry actors, politicians, and think tanks sowing doubt and inhibiting action (230). Developing communication strategies that focus on local and visible issues in and near aquatic environments may be useful in motivating action on issues that indirectly benefit the fight to address climate change causes and effects (231, 232). This is where investments in marine conservation communication have the potential to make a difference.

Many problems in the marine environment are connected to or affected by climate change in some way (233), though they are often unfamiliar to audiences (162) and can be harder to communicate than terrestrial topics. Ocean acidification, for example, is referred to as the evil twin of climate change but is largely invisible to those who do not engage in shellfish harvesting (234), and the risk of mass extinction in the marine environment due to climate change is also a significant threat to human societies but on a longer time scale (235). With limited research focused on marine conservation communication and the knowledge and public perceptions of ocean health (236, 237), it is also challenging to know which findings from EC research can or should be applied and in what context(s). For example, in Malta, DeBono et al. (238) found that perceptions of climate threats to lives and livelihoods are strong drivers for climate policy support and a motivation to act, but does the same hold true for issues specific to aquatic ecosystems? Since the literature on marine conservation communication and public perceptions of ocean health is nascent (237), these topics deserve dedicated scholarly attention.

Even with decades of research from which to draw, many applications of EC do not appear to be rooted in evidence and are often designed to mimic successful or appealing campaigns carried out by other organizations (239). To increase the likelihood of a communication's success, communicators and the organizations they represent need to have clear objectives, to understand their intended audiences, to craft clear and memorable messages, to decide which media they will use to deliver their messages, and to know how they will measure outcomes and evaluate their efforts [(240), Table 2]. This process of information gathering, decision making, and evaluation can be described as strategic environmental communication (SEC), defined by Liang et al. (239) as the application of “strategic communication and theory to the practice and promotion of pro-environmental behaviors [and causes]” (p. 137). SEC differs from strategic communication in that it is done in service of a social cause rather than in service of an organization (239) and has four foundational components that are interrelated: audience, message, media and channels, and measurement and evaluation.

**Table 2:** Evidence-based practices for environmental communication.

**Category**	**Best practices**
**Audiences**	
	Identify audience segments based on common identity factors
	Explore audience knowledge, perceptions, and needs as part of the planning process
	Seek to tell audiences what they need to hear rather than what you want to say
**Messages**	
	Choose and emphasize local issues, recognizable places, and shorter timelines
	Pair problems with possible solutions
	Connect environmental solutions to social systems
	Incorporate scientific facts into compelling narratives
	Inoculate against misinformation
	Avoid the extremes
	Choose trusted messengers to deliver key messages
	Find common ground with audiences
	Tell human stories where possible
	Balance emotional moments
	Show audiences how they can help
	Leverage social norms
	Be selective about the use of imagery
	Design simple messages that are easy to remember
	Repeat messages often
**Media and channels**	
	Share or host content from trusted, verifiable sources
	Select appropriate media and repurpose content for each channel
	Create the highest possible quality media with the resources available to you
	Experiment with different forms of media and storytelling methods
**Measurement and evaluation**	
	Set clear, measurable goals as part of the planning process
	Seek qualitative and quantitative feedback from internal and external stakeholders
	Build reflection into practice

### Audiences

While many environmental communication campaigns are intended for a general public–people who are not trained in the sciences–this is a losing strategy (241). Audiences are not passive recipients of information and groups of people are not homogenous in their worldviews, experiences, or identities. Instead, individuals are active participants in the construction of meaning, a process that is greatly influenced by psychosocial factors (242), including life experiences, and social and cultural norms (236, 243–246). It is for this reason that a message that resonates with one group may generate negative emotions in another (247). For environmental communication to be effective and avoid unintended or undesired consequences like ecophobia or boomerang effect, intentional audience research and segmentation is of critical importance and can determine the success or failure of a campaign (242, 248, 249).

Audience segmentation–a structured process that divides a population into “relatively homogeneous, mutually exclusive subgroupings” [(250), p. 442]–is a stronger approach to communication design and scholarship than those that do not methodically consider audience attributes. It includes three key steps: (1) identifying the population of interest and defining the traits that are relevant to the campaign; (2) collecting and analyzing data on the population of interest; and, (3) grouping individuals with similar characteristics into smaller, more homogeneous groups called audience segments. While segmentation analyses often vary in the methods or data used and have inherent limitations, they are of growing interest in the context of science and environmental communication (242).

Audience segmentation not only provides information about who members of a group are, but can uncover what it is they need to hear, from whom, and how. In addition to supporting the effectiveness of a communication campaign, audience segmentation can help practitioners make the best use of their resources. As such, it is important to dedicate time and attention to audience research. A notable example of audience segmentation in environmental communication is the longitudinal Climate Change in the American Mind survey, which was launched in 2008 by researchers from George Mason and Yale universities (251). Known as Global Warming's Six Americas, this research has led to the creation of six typologies or audience segments that are based on belief in and concern for climate change–ranging from alarmed (most concerned) to dismissive (least concerned) (251). Not only have scholars been able to define each of the six segments and track changes in climate change beliefs over time using this bi-annual survey, but they have also learned more about each of the six audience typologies based on more nuanced dimensions such as health risk perception (252), race and ethnicity (253), and religion (254). These kinds of data and analysis can inform decision making, especially about communication projects and their goals. A campaign designed to increase climate change belief would not do well to dedicate resources trying to reach audience segments that already believe in climate change and support policy actions, just as a campaign designed to encourage political action would not do well in trying to activate the audience segments that are doubtful or dismissive of climate science or the role of government in acting to address its causes and effects. Given the variety of topics, geographies, and sociopolitical systems involved, audience segmentation and analysis can provide valuable insights throughout the communication planning process involved in making OHH science actionable.

### Messages

The content or information packaged for mediated delivery to an audience is called a message. Messages both influence and are the result of strategic development that includes framing, audience research, messenger and media selection, and consideration of externalities like messages from opposing viewpoints (255). Communicators design messages with the goal of creating a shared meaning or set of meanings with recipients through any combination of elements like spoken or written language, still or moving images, and other symbolic elements like music. Message development and research includes strategies like framing (256), selection of trusted messengers (257), narrative design (258–261), visualization and photography (164, 262, 263), and even more granular details like the grammatical choices in a written text (264), the use of humor and other emotional frames (265, 266), or the musical selection in a short-form video (267). While much goes into the design of messages, the most effective ones are simple, clear, memorable, and repeated often by messengers the audience trusts (257, 268, 269).

Messaging research often investigates how audience groups respond to messages, whether as a whole text or by testing individual variables. Framing is a commonly studied component of messaging in the context of environmental communication and involves the strategic ways in which communicators present information to audiences (270–272). While there are a number of frames from which to choose (256), health framing is of growing interest and prevalence in EC (273) as research suggests it can be an effective tool to engage and motivate some audiences to care and act upon environmental issues, with the caveat that this approach should include solution-oriented information to be successful (23, 274). Maibach et al. (22) have also found that audiences respond more positively to messaging that highlights the health benefits associated with policy decisions rather than focusing on associated hazards and risks, which aligns with other research that acknowledges the cognitive costs of emotional appeals related to environmental messages and audiences' needs for information about social rewards and individual efficacy (275). Often studied and applied in health-care settings to achieve behavior change with generally positive outcomes (276, 277), narrative interventions and analyses of narrative elements are also of interest to environmental communication research (258, 259, 278), especially since narratives can appeal to emotions and motivate action by supplementing quantitative environmental data (261).

### Media and channels

Messages are delivered to their audiences using media or channels which can include, but are not limited to, print, video, photography, or spoken word. As society grapples with existential environmental issues from the local to international scales, traditional media–including documentary film, experiential advertising, and news media reporting–are now used in conjunction with digital media like short-form social media films, digital media advocacy campaigns, and virtual or augmented reality (217). The communication tools and channels available to today's environmental communicators are evolving nearly as rapidly as the science that environmental communicators aim to package and distribute to audiences. Many of these modern channels are readily available in some form for anyone with a desire to create and distribute information–accurate or not–in an instant.

During media selection, communicators decide on the ways they intend to package and deliver their message to their intended audience. In the current media landscape, there are many options from which communicators can choose, including print, broadcast, and digital media formats, as well as face-to-face communication (141), exhibits, educational interpretations, and interactive experiences. Factors like audience preferences and the type of messaging to be shared influence which media best support the communication. For example, instrumental music can complement the images displayed in a short form video on an environmental topic and prompt viewers to experience a particular emotion (267), but it is not possible to include instrumental music in a print product like a brochure or a book. Similarly, plastics pollution, oil spills, and natural disasters work well in photographs and other forms of visual media (279), while less visible or longer-term outcomes like ocean acidification or changes in vital ocean currents are much harder to capture with the same methods. Campaigns are not limited to one medium or channel, and many leverage multiple media types to reach different audiences or to reinforce messages in different ways. It is important to note, however, that more complex or technologically advanced forms of communication are not necessarily more effective than simpler ones and may actually undermine a campaign's effectiveness as the messages (12).

Given the complexity of environmental topics and the uncertainty surrounding them, as well as limited resources and budgets, media selection is a critically important decision to make as part of the strategic communication planning process (280–282). Additionally, generalists–or even freelancers–have, for the most part, replaced dedicated environmental journalists, whose jobs were to report on environmental science. Productivity expectations for journalists in the digital era require writers to work on multiple stories under increasingly compressed deadlines, creating other barriers to earning coverage for OHH topics (202, 283). An understanding of the modern media landscape–especially by the institution of science and experts in the field (284)–is essential to the success of any effort in environmental communication.

### Measurement and evaluation

Operationalizing and measuring outcomes of communication are important steps in knowing what works, the degree to which it works, and the context in which it worked. Like a research question informs the methods used in a study, the objective of a communications effort informs how one defines and measures success. Oftentimes, these goals and performance metrics relate to the degree to which a campaign supports an organization's mission (285). While evaluation in the context of strategic communication is not standardized and often narrowly focuses on activities and outputs (e.g., social media impressions, clicks through to links embedded in an email marketing campaign, the financial returns related to a campaign), there have been advances in frameworks and approaches to the practice that have the potential to make planning, implementation, and evaluation of communications more effective (286–288).

Notably, there are calls to move away from one-way communication and measurement in favor of a more open and adaptive approach that considers both quantitative and qualitative information from internal and external stakeholders (289). In these cases, evaluators might reflect on the communication design process and suggest changes an organization can apply moving forward or conduct reception studies to learn more about how audiences construct meaning from campaigns. Despite there being many ways to go about measuring and evaluating strategic communications efforts and an agreement that it is an essential part of communications practice, it is a resource intensive process and one that is often skipped (286).

### Remaining challenges

Like OHH, there are persistent challenges facing EC scholars and practitioners. Many of them relate to tying knowledge to action and making environmental problems relevant and important to audiences to inspire action. Environmental communication scholars and practitioners continue to explore ways to address these challenges that include drawing attention to complex environmental issues and their solutions, combating misinformation and media selection, and supporting efforts to make environmentalism more diverse, equitable, inclusive, and accessible.

Environmental communicators conduct their work in the same places as other strategic communicators, meaning they compete with other organizations and newsworthy events for the attention of their audiences, making it harder and that much more important to draw attention to environmental stories and inspiring the urgency needed to create the social, behavioral, and political changes that can solve them. One barrier is the mismatch between the speed of news cycles and the slow development of environmental problems, which poses challenges for getting past media gatekeepers and earning coverage (290). Another is that of public perceptions; non-expert audiences certainly know that aquatic systems are under threat, but at the same time feel unfamiliar with these environments, perceive distance between ocean issues and daily life, and don't hear enough about these topics to know what exactly is wrong and how they can help (159, 162, 291). Other social headwinds include a lack of familiarity with the growing body of psychological research on topics in the environment and sustainability (240), debates about the role of scientists and other experts in the policy realm (227, 229), a disconnect between the issues that are most pressing to the scientific community and those that receive the most public attention and resources (292), and the fact that, despite wanting to do better, reporters need more opportunities to learn from scientists about pressing issues in the marine environment to cover them better for their audiences (12).

While counter campaigns are nothing new, communicators have an incredible amount of control over their channels of choice in today's media landscape, which can lead to echo chambers that reinforce existing beliefs and introduce people to misinformation, disinformation, and malinformation (293–296). These activities can undermine factual messaging and lead to behavior that inhibits action, such as underestimating public support for climate policy (297). There are, however, five common categories of climate misinformation –that the problem is not real; the problem is not harmful or serious; people do not have a role in creating or contributing to the problem; society cannot do anything to avoid the worst outcomes; and experts in the field do not agree on the facts (298)–and strategies used to deliver it to audiences (203). These positions and strategies have not changed much since the early days of climate denial campaigns, making them fairly easy to identify (203). While there are ways to teach people to spot misinformation about climate and other environmental topics, false beliefs can take hold quickly with individuals, become politicized, and are much harder to counter than proactive efforts (203, 299, 300). Issues related to OHH and the actions to solve them through policy and other means certainly have the potential to be politicized due to cognitive biases like motivated reasoning (301), confirmation bias (302), and hyperbolic discounting (303) and, as a result, subject to many of these same challenges (162).

Environmental communication is directly related to environmental justice. Public communication and participatory processes have the power to shape conversations in ways that devalue the conditions and voices of disempowered groups (224) or don't consider the role of inequities–environmental or otherwise–in shaping local climate narratives, peoples' lived experiences, and policy decisions (177). Issues of representation, power, justice, and accessibility not only play out in the courtroom but make their way into environmental media–if environmental justice topics receive coverage at all (304). Through framing, gatekeeping, and other communication processes, certain voices are amplified and others are muted; sometimes intentionally, sometimes not. Mainstream media often overlooks the causes and histories of environmental injustices and recognizes and reports the positions of institutions like businesses, non-profits, and governments rather than those of citizen advocates (305, 306). Even documentary films that are intended to be ethnographic and objective can reinforce inequities or serve the interests of the filmmaker and the audiences to which they share their art (307). While there are efforts to address environmental justice issues using alternative pathways–such as critical interruptions and storytelling (308, 309), community-centered interventions like photovoice (310, 311), and adopting processes that make room for traditional and indigenous knowledge as important parts of decision making processes (312, 313)–much more needs to be done to make sure environmental communicators are challenging dominant discourses and carrying out their duty of care to serve marginalized communities.

While environmental communication has advanced a great deal as a field of study and a professional practice, more needs to be done to create a body of knowledge to solve an incredible range of complex environmental problems and invites people of all ways of life to get involved, especially with topics not directly related to global climate change.

## Opportunities for collaboration and innovation

Communication is an essential part of the social processes through which OHH science becomes actionable. It is an exciting and opportune time to explore ways to make invisible challenges visible to non-experts and to achieve meaningful progress for the field through collaborative scholarship and practice. But what exactly could communication-focused collaboration look like? I propose five starting points based on the strengths and limitations of these distinct yet complementary fields: (1) definitions and messaging, (2) proactive communication design and evaluation, (3) a shared commitment to environmental justice, (4) bridging science and storytelling, and, (5) training & boundary spanning. These actions stand to support and advance existing efforts to draw attention to ocean ecosystems and its role in our lives, helping to increase marine citizenship worldwide.

By working toward a clearer working definition of ocean and human health, there are opportunities for collaborations across disciplines and sectors to get the messaging right on the science, solutions, actions, and outcomes associated with these topics. Work done on the science and communication of nature-based solutions (NbS) as responses to climate change and biodiversity loss can serve as an important starting point. Seddon et al. (314) found that a lack of consensus and loose definitions of NbS compared to the complex science and social factors behind them can lead to unintended consequences like undermining the urgent need to reduce fossil fuel consumption, over-emphasizing forests and tree planting for carbon sequestration, and overlooking the needs of native ecosystems or the resource rights of local communities, particularly Indigenous peoples. Placing emphasis on nature-health linkages also comes with it the risk of reinforcing dominant discourses of nature as a service provider or sustainability efforts as a conduit for continued economic growth, discourses that ignore the importance of intrinsic valuations and the cultural and personal meanings of nature and place (315, 316). OHH communications should be carefully designed to avoid similar pitfalls, and understand that local efforts can support more unified global messages.

Another opportunity for communication-focused collaboration involves the development, implementation, and evaluation of potential communication strategies with an OHH focus and sharing that knowledge widely. Knowing that individuals experience social-cognitive limitations when receiving information about aquatic ecosystems and knowing that certain issues or solutions in the context of OHH science may be politicized in the future, scholars and practitioners alike can contribute to a proactive and adaptive strategies that are tailored for audiences of interest, to address challenges specific to a particular geographic region, or that focus on solving similar problems in the marine environment across geographic regions. One way to do this is to assess or analyze relevant content that already exists to develop a baseline understanding of topics in OHH that have received attention, how they are communicated, and to whom they appeal. Another involves designing and testing communication interventions to connect local OHH stories to global scientific knowledge or challenges, documenting and sharing results so others may learn from and apply them.

With stated commitments to environmental justice and equity, contributors to OHH and to EC have a responsibility to help make good on these promises, whether working in science, policy, public health, communication, or otherwise. This is especially true as environmental decision-making is not apolitical and decisions inevitably create winners and losers of varying degrees. Community engagement, co-production of knowledge, and incorporation of knowledge beyond the empirical are all activities that require strong communication and an ability to engage many perspectives and lived experiences. So, too, do building and investing in relationships with media gatekeepers in attempts to amplify unheard or underheard voices in mainstream media while also leveraging less traditional channels to make information about environmental justice accessible. Engaged scholarship (224) that investigates environmental injustices and attempts to solve them [e.g., (309, 317)], that shares lessons learned from collaborative efforts that are inclusive of non-empirical knowledge [e.g., (112)], or that examines the effects of environmental changes or solutions to them on marginalized communities (318) can help OHH science and policy advance in ways that are equitable, inclusive, and just. Additionally, there is a great need to continue advancing research beyond the borders of Western, white nations and to incorporate forms of knowledge beyond the empirical, all in service of environmental justice and equitable health outcomes for the global community.

There is an increasing need for environmental professionals who are trained to transcend boundaries and work with stakeholders from different sectors, organizations, and perspectives (319–321). This kind of training can come from interdisciplinary or professional degree programs, novel research collaborations, work experience, professional development, or some combination of education and work experience. Making OHH actionable requires a range of professionals who can understand and facilitate conversations between environmental scientists, social and behavioral scientists, health practitioners, decision makers, community members, and communication professionals, among others (322). Activities these individuals may take on as part of their work could include connecting scientists with reporters in an effort to create more accurate and compelling coverage of the marine environment; teaching health practitioners how to talk to their patients about the environment and human health; and translating the work of experts into accessible and understandable communication campaigns for non-expert audiences. Additionally, the scientific community can encourage participation in these kinds of efforts by moving away from career development paths that are focused on publications, presentations, and teaching (323) and toward a model of career development that also recognizes and celebrates achievements in policy, communication, and community engagement. Degree programs, workplaces, and funders can and should support the development and hiring of more boundary-spanners who are prepared to bring diverse groups together in pursuit of positive change.

Empirical data and storytelling in its many forms can and should be used in support of one another in the context of OHH and can serve to connect local, visible issues or opportunities to ones at a regional, national, or global scale. OHH research covers a range of unique and sometimes unexpected topics, which creates opportunities to tell stories that appeal to audiences of all kinds. For audiences who are invested in biodiversity conservation, stories about animals may be enough. For parents of young children, stories of local air and water quality may be a better choice. For seafood producers, consumers, and business-minded individuals, stories about local fisheries, their quality, and their value may work well. For people who are interested in physical, psychological, or spiritual wellness, stories about athletes, recreation opportunities, or people who have otherwise benefitted from aquatic ecosystems may be appealing. Whatever the topic–be it cats as sentinel species for ocean quality (34) or the story of a successful partnership between scientists and Indigenous communities related to harmful algal blooms (112)–there are many ways to tell these important and memorable stories and inspire action.

## Conclusion

To advance the findings of ocean and human health research and environmental communication and create positive outcomes for the billions of individuals who live on our ocean-planet, we need to move beyond the production of knowledge and begin testing how best to use it to advance conservation and sustainability goals. This is especially true if we want the Ocean Decade to be one of meaningful and lasting change, rather than a symbolic global pledge. With a broad base of research available in OHH science and environmental communication, it is time to bridge the two in focused research and professional practice that can inform strategies that inspire social and behavioral changes that benefit aquatic systems and the communities that rely on them. It is no simple task, though for those who are willing to work on it over the next decade and beyond, the potential rewards are significant.

## Author contributions

The author confirms being the sole contributor of this work and has approved it for publication.

## Conflict of interest

The author declares that the research was conducted in the absence of any commercial or financial relationships that could be construed as a potential conflict of interest.

## Publisher's note

All claims expressed in this article are solely those of the authors and do not necessarily represent those of their affiliated organizations, or those of the publisher, the editors and the reviewers. Any product that may be evaluated in this article, or claim that may be made by its manufacturer, is not guaranteed or endorsed by the publisher.
